# Comparative effectiveness of FAST combined with MRI and CT in wound evaluation of blunt abdominal trauma

**DOI:** 10.3389/fmed.2025.1702765

**Published:** 2026-01-13

**Authors:** Fugang Chen, Yu Wang, Xiaoqiang Liu, Zhaoyun Wang, Xin Li, Xueshuang Tang, Jun Gao

**Affiliations:** 1 Department of Radiology, The Second Hospital of Dalian Medical University, Dalian, Liaoning, China; 2 Department of Ultrasound, The Second Hospital of Dalian Medical University, Dalian, Liaoning, China; 3 Department of Wound Healing, The Second Hospital of Dalian Medical University, Dalian, Liaoning, China

**Keywords:** accuracy, blunt abdominal trauma, CT, diagnostic time, MRI, ultrasonic evaluation method of trauma

## Abstract

**Background:**

Blunt abdominal trauma, often resulting from motor vehicle collisions (MVCs), assaults, recreational accidents, or falls, is commonly assessed in emergency departments using the focused assessment with sonography for trauma (FAST). FAST offers advantages such as ease of use and rapid identification for intra-abdominal free fluid, an indirect marker of visceral injury. However, its diagnostic performance is highly dependent on operator expertise, limiting its ability to accurately characterize the type and severity of injuries.

**Objective:**

This study aimed to assess the diagnostic performance of FAST combined with magnetic resonance imaging (MRI) compared to computed tomography (CT) in the evaluation of hemodynamically stable patients with blunt abdominal trauma.

**Methods:**

A retrospective, single-center study was conducted involving 87 patients admitted between February 2018 and September 2023 with suspected blunt abdominal trauma. Only hemodynamically stable patients were included in this study. All patients were underwent FAST, MRI, and CT imaging, with surgical or pathological findings serving as the gold standard. The diagnostic accuracy, localization capability, and time to diagnosis were between the FAST + MRI and CT.

**Results:**

Among the 87 patients, 36 were confirmed to have abdominal organ injuries. The FAST + MRI approach identified 34 true-positive cases, whereas CT identified 30. FAST + MRI showed significantly higher diagnostic accuracy and localization capability compared to CT (*p* < 0.05). Additionally, for intra-abdominal cavity injuries, 18 cases were accurately diagnosed using FAST + MRI, compared to 12 with CT.

**Conclusion:**

Our findings indicate that in hemodynamically stable patients, the combination of FAST and MRI provides superior diagnostic accuracy and localization for blunt abdominal trauma compared to CT, with timely diagnostic efficiency. These findings suggest that FAST + MRI may serve as a useful complementary imaging strategy in hemodynamically stable patients; however, further prospective studies comparing this approach to contrast-enhanced CT and incorporating patient outcome measures are warranted.

## 1 Introduction

With the rapid development of transportation, construction, industry and agriculture, the incidence and severity of blunt (closed) abdominal trauma have risen markedly (1, 2). Recent estimates indicate that the mortality from this injury pattern is up to 20-fold higher in developed than in developed countries, and that roughly one-third of deaths are potentially preventable through timely diagnosis and intervention (3, 4). Compared with cranial, orthopedic, or thoracic injuries, abdominal trauma is distinguished by its complexity, urgency, and propensity for atypical or masked clinical signs, which together account for high rates of missed or delayed diagnoses (5, 6). Computed tomography (CT) remains the cornerstone of diagnostic work-up because of its rapid image acquisition and high spatial resolution, allowing precise delineation of injury location, extent, and related complications information that is critical for selecting operative versus non-operative management. Nonetheless, CT exposes patients to ionizing radiation and iodinated contrast, posing risks of radiation-induced malignancy and contrast allergy or nephropathy (7). Focused assessment with sonography for trauma (FAST) offers a fast, non-invasive, and inexpensive alternative for detecting free intraperitoneal fluid, an indirect marker of visceral injury, in the resuscitation bay. Its diagnostic yield, however, is highly operator-dependent and can be compromised by body habitus or bowel gas (8). Magnetic resonance imaging (MRI) provides superior soft-tissue contrast without ionizing radiation or iodinated contrast, enabling accurate characterization of parenchymal and vascular injuries. Yet its longer acquisition time, higher cost, and need for patient cooperation limit widespread emergency use (9). Various imaging modes have been applied to the diagnosis of blunt abdominal trauma.

Current trauma typically employs CT following an initial FAST to confirm or exclude intra-abdominal injury. The present study explores whether combining FAST with MRI could improve diagnostic accuracy, particularly soft-tissue injuries compared to CT alone. We do not propose replacing CT; rather, we aim to determine whether FAST + MRI constitutes a valuable complementary strategy in hemodynamically stable patients, especially when CT is equivocal, contraindicated, or unavailable. Therefore, this study aimed to assess the diagnostic performance of FAST combined with magnetic resonance imaging (MRI) compared to computed tomography (CT) in the evaluation of hemodynamically stable patients with blunt abdominal trauma.

## 2 Research objects and methods

### 2.1 Sample size calculation

The sample size was calculated using the formula n = [*Z*^2^·*p*·(1 − *p*)]/*d*^2^, where *Z* = 1.96 (for a 95% confidence interval), *d* = 0.05 (margin of error), and the expected sensitivity (*p*) was set at 94%. This value was based on prior research (10), which reported an overall diagnostic accuracy of 93.33% and a sensitivity of 92.8% for CT in detecting blunt abdominal trauma. To ensure a conservative yet robust estimation and to reflect our hypothesis that FAST + MRI offers clinically meaningful superiority, we assumed an expected sensitivity of 94%. Based on this parameter, the calculated sample size was 86 patients; this number was rounded up to 87 to ensure adequate statistical power.

### 2.2 Inclusion and exclusion criteria

#### 2.2.1 Inclusion criteria

Confirmed blunt abdominal trauma verified intra-operatively or by pathological examination.

1. Age ≥18 years.
2. Injury Severity Score (ISS) ≥16, indicating moderate-to-severe trauma, as per the standard trauma classification criteria. This threshold ensured the inclusion of patients with clinically meaningful injuries while still allowing the selection of those who were hemodynamically stable enough to safely undergo MRI, which was a requirement for protocol consistency.
3. Complete imaging triad: FAST, MRI, and CT performed within a ≤60-min interval to minimize interval clinical change and ensure temporal comparability. This criterion ensured temporal comparability between imaging results while minimizing clinical changes between modalities. These examinations were performed under a research protocol, and only clinically stable patients able to undergo all three modalities without delay were included.
4. Only hemodynamically stable patients were included in this study, defined as those with a systolic blood pressure ≥90 mmHg, heart rate ≤100 beats/min, and without clinical or laboratory evidence of ongoing shock or active bleeding at presentation. This definition ensured objective consistency in determining vital stability across all included participants.
5. Comprehensive clinical data available for analysis.
6. Written informed consent obtained from the patient or a legal surrogate.

#### 2.2.2 Exclusion criteria

1. Open (penetrating) abdominal trauma.
2. Cardiopulmonary resuscitation in progress at the time of screening.
3. Pre-existing abdominal conditions that could confound image interpretation or mimic traumatic lesions, such as extensive abdominal adhesions, large abdominal masses (e.g., hepatomegaly, splenomegaly, tumors), or chronic inflammatory conditions (e.g., Crohn’s disease, tuberculosis peritonitis).
4. Delayed presentation: interval from injury to initial clinical evaluation >4 h, given evidence that delayed imaging substantially alters traumatic findings. This time limit was chosen based on prior clinical evidence suggesting that delayed imaging beyond 4 h may significantly alter trauma manifestations, including bleeding evolution, organ displacement, or secondary complications, thus confounding diagnostic consistency between modalities.
5. Coagulopathy, defined as international normalized ratio (INR) >1.5, platelet count <50,000/μL, or activated partial thromboplastin time (aPTT) >40 s. These patients were excluded because coagulopathy increases the risk of ongoing hemorrhage and hemodynamic instability and may alter the imaging manifestations of traumatic bleeding, thereby introducing heterogeneity that could confound comparison of diagnostic performance between imaging modalities in a stable cohort.
6. Concurrent major injuries to the head, chest, or extremities that may interfere with diagnostic imaging timing, interpretation, or clinical management prioritization.

### 2.3 Grouping

Clinical data from 87 patients with confirmed blunt (closed) abdominal trauma who were admitted to our institution between February 2018 and September 2023 were retrospectively analyzed. All enrolled patients underwent FAST, MRI, and CT imaging. Final diagnosis were established using surgery or pathological findings, or, when surgery was not performed, through consistent clinical and imaging follow-up, which served as the gold standard. Prior to MRI, all patients were pre-screened by the emergency department team for potential contraindications, such as metallic implants, pacemakers, or claustrophobia. Only clinically stable patients without contraindications and with sufficient ability to cooperate with MRI procedures were included.

**Figure 1:**
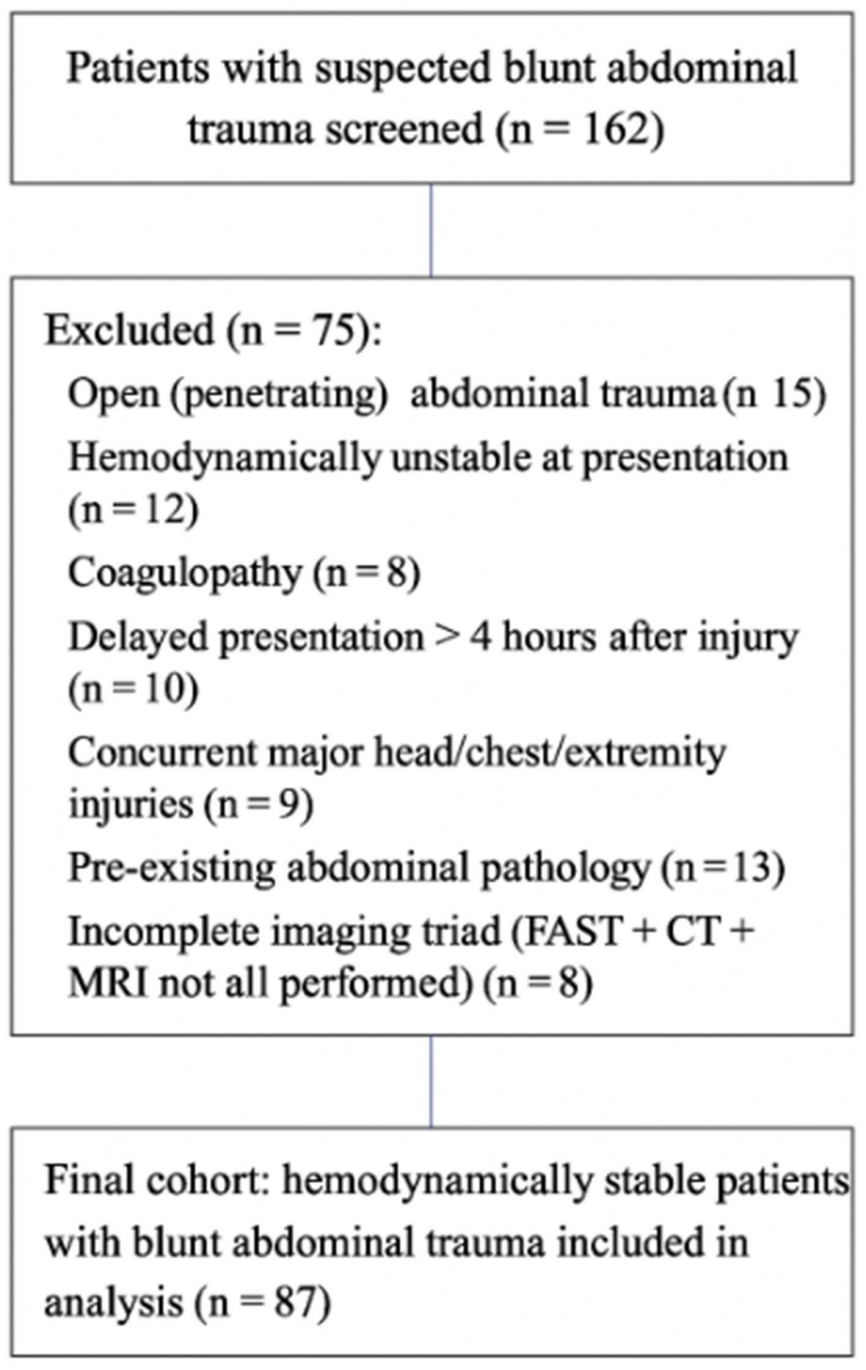
Flow diagram showing patient selection and exclusion process. Of 162 patients screened, 75 were excluded for the following reasons: penetrating abdominal trauma (*n* = 15), hemodynamic instability at presentation (*n* = 12), coagulopathy (*n* = 8), delayed presentation >4 h (*n* = 10), concurrent major injuries (*n* = 9), pre-existing abdominal pathology (*n* = 13), and incomplete imaging triad (*n* = 8). The remaining 87 hemodynamically stable patients formed the final study cohort.

Patients were categorized into two groups based on the imaging modality combinations used: the FAST + MRI group, consisting of patients who underwent FAST combined with MRI, and the FAST + CT group, comprising patients evaluated with FAST followed by CT. In clinical practice at our center, all patients first underwent FAST, followed by either MRI or CT; therefore, the FAST + CT group refers to patients evaluated with FAST followed by CT. All 87 hemodynamically stable patients underwent FAST, MRI, and CT within 60 min of each other, allowing for direct comparison of diagnostic performance across the same cohort for both FAST + MRI and FAST + CT combinations. This information was previously presented as Figure 1 but has now been incorporated into the text to improve clarity and readability. Baseline characteristics of the study population, including age, sex distribution, mechanism of injury, time from injury to hospital admission, vital signs at presentation, and GCS scores, are summarized in Table 1. The patient inclusion and exclusion process is illustrated in Figure 1.

**Table 1:** Baseline characteristics of hemodynamically stable patients with blunt abdominal trauma (*n* = 87).

Characteristic	Value
Total number of patients	87
Age, years, mean ± SD	36.72 ± 5.3
Male, *n* (%)	59 (67.8%)
Female, *n* (%)	28 (32.2%)
Motor vehicle collision/traffic accident, *n* (%)	47 (54.0%)
Fall, *n* (%)	22 (25.3%)
Blunt force trauma, *n* (%)	18 (20.7%)
Time from injury to admission, hours, mean ± SD	1.61 ± 0.2
Injury Severity Score (ISS), mean ± SD (range)	21.4 ± 3.2 (16–30)
Hemodynamic status at presentation	All patients hemodynamically stable
Systolic blood pressure, mmHg, mean ± SD	124.5 ± 9.3
Heart rate, beats/min, mean ± SD	86.2 ± 11.1
Respiratory rate, breaths/min, mean ± SD	18.7 ± 2.9
Glasgow Coma Scale (GCS) score, mean ± SD	14.9 ± 0.4

Gold standard: surgical or pathological confirmation when available; otherwise concordant follow-up imaging and clinical assessment.

The study protocol was approved by the Ethics Committee of the Second Hospital of Dalian Medical University (Approval No. SD202401013). Written informed consents were obtained from all participants or their legally authorized representatives prior to enrollment. MRI was not selectively performed based on clinical judgment or necessity; instead, it was uniformly conducted for all enrolled patients who met the inclusion criteria, were hemodynamically stable, and had no contraindications to MRI. This protocol enabled a direct and controlled comparison of the diagnostic performance of FAST, MRI, and CT. Patients who were clinically unstable or had contraindications to MRI were excluded from the study to maintain methodological consistency. A total of 29 patients were excluded based on the predefined exclusion criteria, resulting in a final cohort of 87 patients who were included in the final analysis. Baseline demographic and clinical characteristics of the study cohort are presented in the Results section (Table 1).

### 2.4 CT examination

The patient was positioned supine for the CT scan. A Siemens 16-row CT scanner was used to image from the top of the diaphragm to the lower pole of the right kidney and then down to the pelvic cavity, based on the patient’s condition. The parameters for the CT plain scan were as follows: tube current 200 mA, tube voltage 120–140 kV, pitch 0.938, field of view 50 × 50 cm, matrix 512 × 512, spiral time 0.8 s, slice thickness 2.5 mm, and reconstruction thickness 1.25 mm.

Contrast-enhanced CT was performed using nonionic iodinated contrast agents unless contraindicated. The contrast phase included portal venous imaging, which is optimal for abdominal trauma assessment. In patients with renal insufficiency or known allergy to iodinated contrast agents, only non-contrast-enhanced CT scans were performed. CT was performed using portal-venous phase contrast enhancement whenever clinically feasible; however, in patients with contraindications, non-contrast CT was performed (18/87 patients, 20.7%). Thus, the CT comparator in this study comprised a mixture of contrast-enhanced and non-enhanced examinations, reflecting real-world clinical constraints.

### 2.5 FAST examination

The patient was positioned supine. The examination was performed using a Siemens ACUSON Sequoia ultrasonic diagnostic system with a 3.5 MHz probe. The probe was used to scan the left and right intercostal spaces, as well as the area above the pubic symphysis, to quickly assess the presence of any echogenic dark areas in the abdominal cavity. All FAST examinations were performed by radiologists from the emergency ultrasound team who had formal training in trauma ultrasound and routinely perform FAST as part of institutional trauma protocols. A standardized FAST protocol (including perihepatic, perisplenic, pelvic, and pericardial views) was used for all patients. Images and cine loops were archived, and examinations with equivocal findings or discrepancies with subsequent imaging were re-evaluated in consensus to ensure quality and consistency.

### 2.6 MRI examination

Prior to MRI scanning, all patients were assessed by emergency physicians for eligibility and safety. Only patients with stable vital signs and no contraindications to MRI, such as metallic implants, pacemakers, or severe claustrophobia, were eligible for MRI scanning. Prior to the examination, all patients were instructed to remove any metallic objects. MRI was performed with the patient positioned supine to ensure optimal image acquisition and patient comfort. A Philips Ingenia 3.0T MRI scanner with an abdominal coil was used. Imaging was focused exclusively on the abdomen and pelvis for trauma assessment; no whole-body or additional regional MRI examinations were performed. Scanning included plain abdominal imaging with the following parameters: T1-weighted imaging with a repetition time of 560 ms and an echo time of 6.8 ms; T2-weighted imaging with a repetition time of 3,460 ms and an echo time of 85.6 ms. The scan settings included a slice thickness of 5 mm, a slice interval of 0.3 mm, a field of view of 38 × 38 cm, and a matrix size of 512 × 512. MRI was conducted without the use of contrast agents. This decision was made to avoid potential adverse reactions and to ensure comparability across all patients. The high tissue contrast of MRI sequences (T1 and T2 weighted) provided sufficient diagnostic detail for injury assessment without contrast enhancement.

To enhance reproducibility, we have now provided full technical details of the MRI acquisition protocol, including scanner type, coil configuration, imaging sequences, repetition time, echo time, slice thickness, interslice gap, field of view, and matrix size. These comprehensive parameters ensure that the MRI acquisition process can be replicated in similar clinical settings. Injuries were evaluated against a reference standard defined as surgical or pathological confirmation when available, or concordant follow-up imaging and clinical assessment in non-operatively managed patients; the distribution of these confirmation methods is reported in the Results section.

### 2.7 Evaluation method

The imaging results from FAST + MRI and CT were independently evaluated by two senior radiologists with extensive experience in abdominal imaging, using a double-blind assessment protocol. These readers were not involved in performing the index FAST examinations and were blinded to each other’s interpretations and to the final clinical outcomes. In cases of diagnostic disagreement, a consensus was reached through joint review and discussion. For the purpose of analysis, organ findings were categorized as either positive (injury present) or negative (no injury). In all analyses, including those presented in Tables 1–3, the reference (“gold standard”) diagnosis was defined as surgical or pathological confirmation when available; for patients managed non-operatively, the gold standard was a final diagnosis based on concordant follow-up imaging and clinical assessment, in accordance with standard trauma care protocols. Surgical or pathological diagnoses served as the gold standard for evaluating diagnostic accuracy. The gold standard diagnosis was determined by surgical or pathological findings when available. For patients managed non-operatively, the final diagnosis was based on clinical follow-up and repeat imaging consistent with the initial diagnosis, in accordance with standard trauma care protocols.

For each imaging modality, organ injuries were evaluated using standardized criteria. Assessment included: (1) presence and estimated volume of intraperitoneal hemorrhage (categorized as minimal, moderate, or large based on distribution and depth measurements); (2) presence of free intra-abdominal gas (pneumoperitoneum); (3) characteristics of solid-organ injury, including contusion, laceration, and intraparenchymal hematoma; and (4) features suggestive of hollow viscus injury, such as bowel wall discontinuity, mural thickening or edema, mesenteric fat stranding, and localized fluid collections. These criteria were applied consistently across FAST, MRI, and CT evaluations.

### 2.8 Observation index

The qualitative diagnosis results of FAST + MRI and CT for abdominal parenchymal organ injuries were observed and recorded. Additionally, diagnostic time for intra-abdominal organ injury was defined as the interval from hospital admission to the completion and interpretation of the imaging study (CT or FAST + MRI) by a senior radiologist. This interval reflects the timeframe within which imaging-based diagnosis were made to guide subsequent clinical decision-making. It does not include time to surgical confirmation, which served solely as the reference standard for evaluating diagnostic accuracy.

### 2.9 Flow chart

The research flow chart is shown in Figure 1.

### 2.10 Statistical method

Statistical analysis was performed using SPSS version 22.0 (IBM Corp., Armonk, NY, United States). Continuous variables conforming to a normal distribution were expressed as mean ± standard deviation ( $\bar{x}\pm s$ ), and comparisons between groups were conducted using the independent samples *t*-test. Categorical variables were presented as counts and percentages, and group comparisons were assessed using the chi-square *χ*^2^ test. Receiver operating characteristic (ROC) curves and the area under receiver (AUC) were used to evaluate the diagnostic performance of each imaging modality. Given the binary nature of diagnostic outcomes, ROC/AUC values were calculated descriptively without formal statistical comparison using the DeLong test. A two-sided *p*-value <0.05 was considered statistically significant.

## 3 Results

### 3.1 Baseline characteristics

A total of 162 patients with blunt abdominal trauma were screened during the study period; 75 were excluded according to the predefined criteria (Figure 1), leaving 87 patients for final analysis. The study cohort comprised 87 hemodynamically stable patients with blunt (closed) abdominal trauma, including 59 males (67.82%) and 28 females (32.18%), with a mean age of 36.72 ± 5.3 years. The primary mechanisms of injury were traffic accidents (54.02%), falls (25.29%), and blunt force trauma (20.69%). The mean time from injury to hospital admission was 1.61 ± 0.2 h. The mean Injury Severity Score (ISS) of the study cohort was 21.4 ± 3.2 (range 16–30), indicating that all enrolled patients sustained moderate-to-severe trauma. At presentation, patients had a mean systolic blood pressure of 124.5 ± 9.3 mmHg, heart rate of 86.2 ± 11.1 beats per minute, respiratory rate of 18.7 ± 2.9 breaths per minute, and a mean Glasgow Coma Scale (GCS) score of 14.9 ± 0.4. Injury confirmation methods included surgical or pathological verification in 28 patients (32.2%) and concordant follow-up imaging with clinical assessment in 59 patients (67.8%). These data are now included in Table 1 for clarity and completeness. Baseline demographic and clinical characteristics are summarized in Table 1.

### 3.2 Comparison of qualitative diagnosis results between FAST + MRI and CT in abdominal parenchymal organ injury

Among the 87 included patients, the reference standard confirmed abdominal parenchymal organ injury in 36 cases and absence of injury in 51 cases. FAST + MRI correctly identified a larger proportion of injured patients than CT (34 vs. 30 true-positive examinations) and produced fewer false-negative and false-positive findings overall. CT missed several injuries that were subsequently detected by FAST + MRI or at surgery, whereas FAST + MRI misclassified fewer patients in both directions. The distribution of true-positive, false-positive, false-negative, and true-negative results for each modality is summarized in Table 4.

### 3.3 Comparison of qualitative diagnostic value of FAST + MRI and CT in abdominal parenchymal organ injury

**Figure 2:**
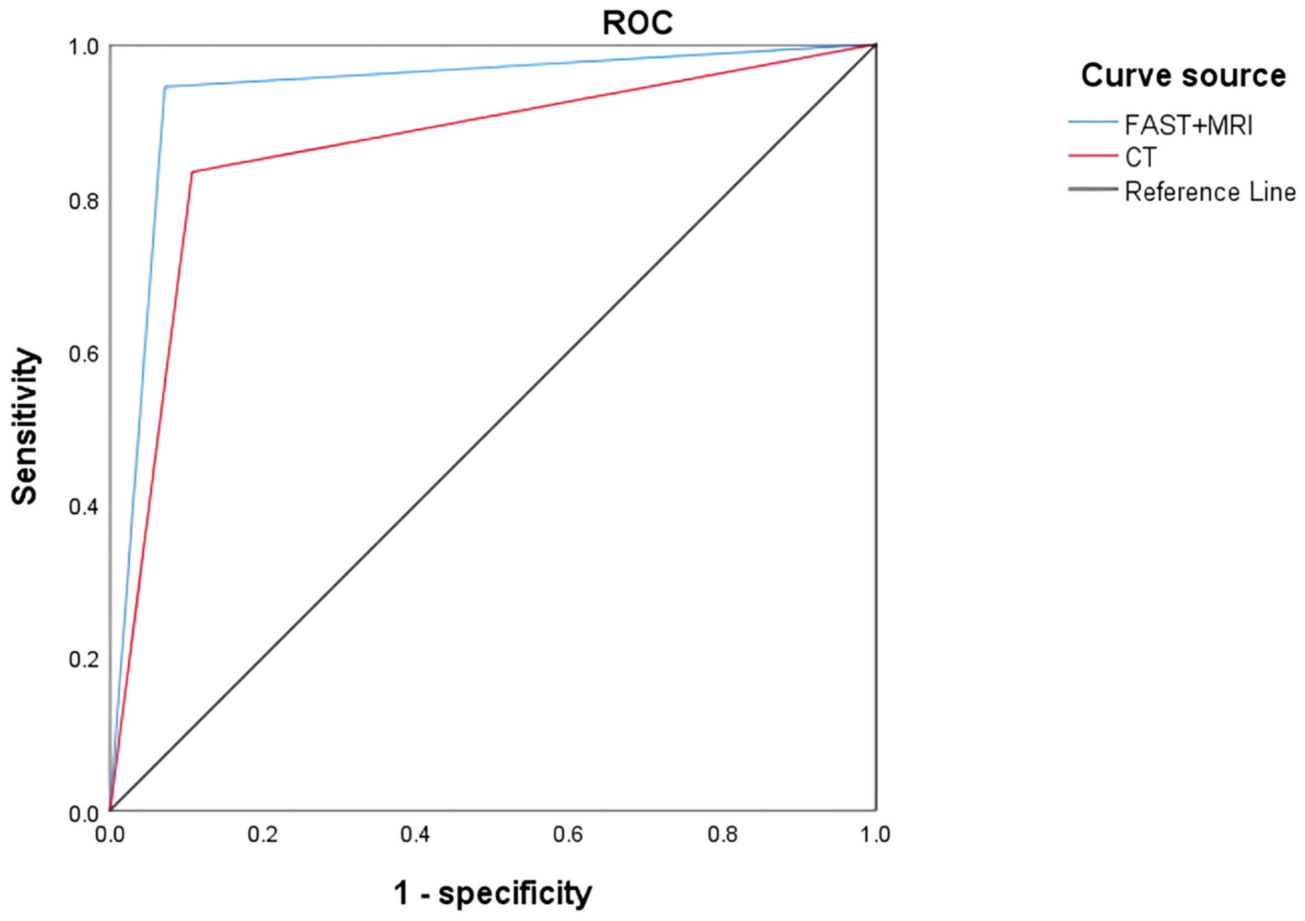
ROC curve of qualitative diagnosis of abdominal parenchymal organ injury by FAST + MRI and CT.

Consistent with these findings, FAST + MRI demonstrated significantly higher overall diagnostic accuracy than CT for the qualitative assessment of abdominal parenchymal organ injury (*p* = 0.036). In this analysis, “qualitative diagnosis” referred to the presence or absence of abdominal parenchymal organ injury as determined by each imaging modality, without formal quantitative grading of hemorrhage or gas volume. Although FAST + MRI yielded numerically higher sensitivity, specificity, positive predictive value, and negative predictive value than CT, these differences did not reach statistical significance, indicating that the main advantage of FAST + MRI lies in its more balanced classification of injured and non-injured patients rather than in any single metric. FAST + MRI also showed stronger agreement with the reference standard, as reflected by a higher Kappa coefficient. The complete set of diagnostic performance parameters for both modalities is presented in Table 2 and illustrated by the ROC curves in Figure 2.

**Table 2:** Comparison of qualitative diagnostic value (injury present vs. absent) of FAST + MRI and CT in abdominal parenchymal organ injury.

Inspection mode	Sensitivity	Specificity	Accuracy rate	Positive predictive value	Negative predictive value	Kappa value
FAST + MRI	94.44	96.08	95.40	94.44	96.08	0.905
CT	83.33	88.24	86.21	83.33	88.24	0.715
*χ* ^2^	2.250	2.170	4.405	2.250	2.170	—
*p*	0.134	0.141	0.036	0.134	0.141	—

Gold standard: surgical or pathological confirmation when available; otherwise concordant follow-up imaging and clinical assessment.

### 3.4 Comparison of FAST + MRI and CT in localization diagnosis of abdominal parenchymal organ injury

**Figure 3:**
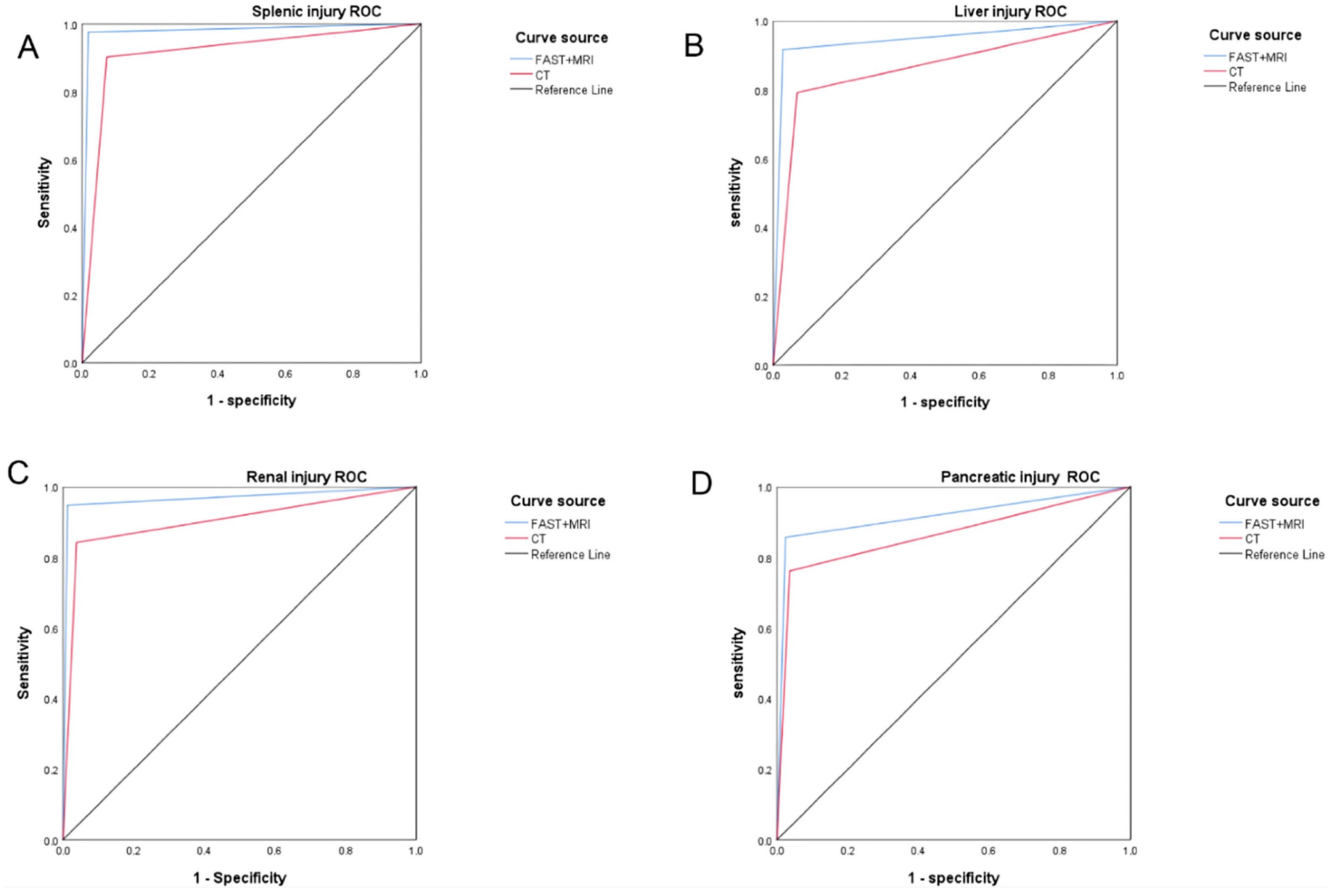
ROC curve of 3FAST + MRI and CT in the localization diagnosis of abdominal parenchymal organ injury: **(A)** spleen injury; **(B)** liver injury; **(C)** renal injury; **(D)** pancreatic injury.

When individual parenchymal organs were analyzed, FAST + MRI again outperformed CT in localization accuracy. Overall, FAST + MRI correctly localized 90 of 96 confirmed organ injuries, compared with 81 of 96 for CT (*p* = 0.037). This advantage was evident across the main solid organs evaluated in this study, including the spleen, liver, kidneys, and pancreas. In particular, FAST + MRI more reliably depicted the extent and exact site of parenchymal disruption, intraparenchymal hematoma, and associated periorgan collections, which facilitated closer correspondence with surgical and pathological findings. Organ-specific values for localization accuracy, sensitivity, specificity, and AUC are detailed in Table 3, and representative ROC curves for each organ are shown in Figure 3.

**Table 3:** Comparison of FAST + MRI and CT in the localization diagnosis of abdominal parenchymal organ injury [*n* (%)].

Wound site	Gold standard	FAST + MRI group	FAST + CT group	*χ* ^2^	*p*
Spleen injury	41	40 (97.56)	37 (90.24)	1.917	0.166
Liver damage	24	22 (91.67)	19 (79.17)	1.505	0.220
Renal injury	19	18 (94.74)	16 (84.21)	1.118	0.209
Pancreatic injury	12	10 (83.33)	9 (75.00)	0.253	0.615
Total	96	90 (93.75)	81 (84.38)	4.331	0.037

Gold standard: surgical or pathological confirmation when available; otherwise concordant follow-up imaging and clinical assessment.

**Table 4:** Comparison between 2FAST + MRI and CT in qualitative diagnosis of abdominal parenchymal organ injury (*n* = 87).

Inspection mode	Positive/Negative	Gold standard		Total
		Positive	Negative	
FAST + MRI group	Positive	34	2	36
	Negative	2	49	51
FAST + CT group	Positive	30	6	36
	Negative	6	45	51
Total	—	36	51	87

**Table 5:** Comparison of FAST + MRI and CT in the localization diagnosis of abdominal cavity organ injury [*n* (%)].

Wound site	Gold standard	FAST + MRI group	CT group	*χ* ^2^	*p*
Stomach and intestines	10	9 (90.00)	7 (70.00)	1.250	0.264
Jejunum and colon	7	6 (85.71)	3 (42.86)	2.800	0.094
Bladder	4	3 (75.00)	2 (50.00)	0.533	0.465
Total	21	18 (85.71)	12 (57.14)	4.200	0.040

**Table 6:** Comparison of diagnostic time between FAST + MRI and CT ( $\bar{x}\pm s$ ).

Inspection mode	Diagnostic time (min)
FAST + MRI group	30.24 ± 3.01
FAST + CT group	28.37 ± 3.04
*t*	1.896
*p*	0.059

### 3.5 Comparison of FAST + MRI and CT in the location diagnosis of abdominal cavity organ injury

**Figure 4:**
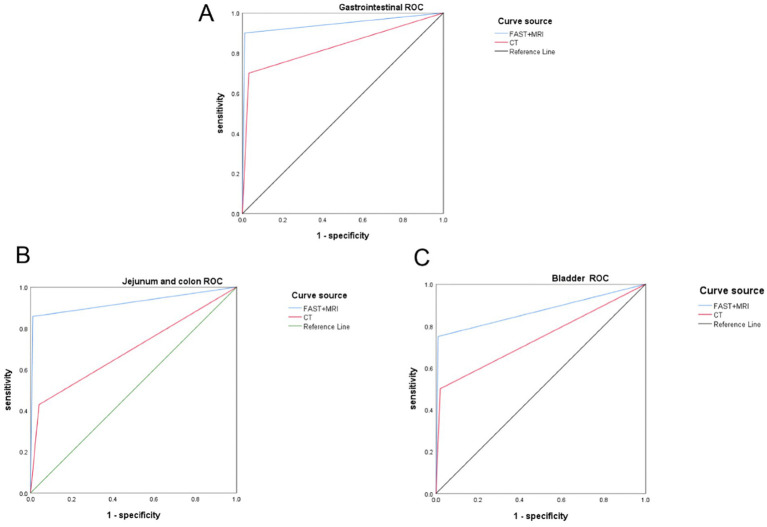
ROC curve of 4FAST + MRI and CT in the localization diagnosis of abdominal cavity organ injury: **(A)** gastrointestinal tract; **(B)** jejunum and colon; **(C)** bladder.

For abdominal cavity organ injuries, such as lesions of the gastrointestinal tract, jejunum and colon, and bladder, FAST + MRI also demonstrated superior diagnostic performance. Of the 21 confirmed abdominal cavity injuries, FAST + MRI correctly identified and localized 18 cases, whereas CT did so in only 12 cases (*p* = 0.040). The benefit of FAST + MRI was most pronounced for subtle hollow-viscus injuries, where CT findings were frequently obscured by gas or bone artifacts and appeared equivocal. In contrast, the higher soft-tissue contrast of MRI allowed clearer visualization of mural disruption, mesenteric changes, and localized fluid collections, while FAST provided rapid functional information and initial suspicion of injury. Overall, the difference in localization accuracy for abdominal cavity organ injuries between FAST + MRI (18/21, 85.71%) and CT (12/21, 57.14%) was statistically significant (*χ*^2^ = 4.200, *p* = 0.040), as shown in Table 5 and Figure 4.

### 3.6 Comparison of diagnostic time between FAST + MRI and CT

The mean diagnostic time was 30.24 ± 3.01 min in the FAST + MRI group and 28.37 ± 3.04 min in the FAST + CT group. Although the FAST + MRI group required slightly more time, the difference was not statistically significant (*t* = 1.896, *p* = 0.059) (Table 6).

### 3.7 Distribution of FAST examination results prior to MRI and CT

Of the 87 patients included in the study, 38 (43.68%) had a positive FAST examination (i.e., free fluid or suspected injury identified). The remaining 49 patients (56.32%) had a negative FAST finding. Regardless of the FAST outcome, all patients underwent both MRI and CT imaging as per the study protocol, ensuring consistency and comparability across imaging modalities.

### 3.8 Patient safety, comorbidities, and hospital outcomes

No patients experienced clinical decompensation during any imaging procedures (FAST, CT, or MRI). All imaging studies were completed without procedure-related adverse events. The in-hospital mortality rate was 0%. The mean length of hospital stay was 9.3 ± 2.7 days. Pre-existing comorbidities were documented in 17 patients (19.5%), including hypertension (*n* = 9), diabetes mellitus (*n* = 5), and coronary artery disease (*n* = 3).

## 4 Discussion

It is crucial to quickly and accurately evaluate the severity of blunt abdominal trauma to guide treatment and prognosis. This assessment involves critical organs such as the liver, spleen, gastrointestinal tract, pancreas, and kidneys.

While CT remains the standard follow-up after FAST in most trauma algorithms, our study investigated whether combining FAST with MRI could improve diagnostic precision, particularly for soft tissue and hollow organ injuries. The FAST + MRI approach was chosen to explore the potential of MRI as a complementary tool to reduce radiation exposure and enhance injury localization, especially in hemodynamically stable patients.

Our findings confirm that while CT remains the conventional imaging standard, combining FAST with MRI enhances diagnostic accuracy and organ localization in hemodynamically stable trauma patients, addressing some of CT’s limitations without additional diagnostic delay.

It is important to acknowledge that this study utilized non-contrast CT as the comparator imaging modality, rather than contrast-enhanced CT, which is widely recognized as the gold standard in trauma imaging, particularly for detection of vascular injuries and active hemorrhage. This methodological choice was guided by institutional protocols and patient safety considerations, especially in cases involving renal insufficiency or contrast allergy. However, this limitation inherently reduces the diagnostic benchmark against which FAST + MRI was evaluated. Consequently, while the findings highlight the potential utility of FAST + MRI in hemodynamically stable patients, the interpretation of these results should be made with caution and in the context of this important limitation.

The results of this study indicate that the qualitative diagnostic accuracy of FAST + MRI is superior to that of CT. This enhanced performance may be attributed to the fact that CT imaging is susceptible to bone-related artifacts, which can impair the accurate evaluation of adjacent soft tissue structures. This observation aligns with the results reported by Sargent et al., who similarly noted the limitations of CT in delineating soft tissue injuries in the presence of osseous interference (11). FAST allows for rapid assessment of abdominal organ movement and blood flow at the bedside, eliminating the need for patient transfer to the radiology suite for CT or MRI. This is particularly advantageous for evaluating the functional state of internal organs. In our study, the sequential use of FAST followed by MRI demonstrated that combining these two modalities provides complementary diagnostic strengths: FAST rapidly detects free fluid and suggests potential injury sites, while MRI confirms and characterizes the nature and extent of these injuries with higher precision. This combined approach improved diagnostic confidence and reduced misclassification rates compared with CT alone, indicating that MRI’s superior soft-tissue contrast effectively compensates for FAST’s limited specificity in non-hemorrhagic or deep-seated injuries. Therefore, the integration of FAST and MRI can serve as a practical, radiation-free diagnostic pathway for hemodynamically stable patients when CT findings are equivocal or contrast use is contraindicated.

The results of this study show that the accuracy of FAST + MRI in locating and diagnosing abdominal parenchymal organ injury is higher than that of CT. The improved diagnostic accuracy of FAST + MRI compared with CT may be explained by their combined diagnostic complementarity rather than imaging mechanics alone. FAST identifies fluid and gross injury patterns rapidly, while MRI clarifies ambiguous or complex lesions with higher anatomical precision (12, 13). This synergy allows for more accurate localization and better alignment with surgical or pathological findings (14). Notably, while the sensitivity and specificity of FAST + MRI did not reach statistical significance compared to CT, the overall accuracy was significantly higher. This discrepancy likely reflects a more balanced performance of FAST + MRI in correctly identifying both true positives and true negatives. In our study, FAST + MRI resulted in fewer total misclassifications (*n* = 4) than CT (*n* = 12), suggesting that the improved accuracy stems from reductions in both false positives and false negatives. This may indicate the complementary strengths of FAST and MRI in identifying different aspects of abdominal organ injury such as hemorrhage, parenchymal disruption, and soft tissue details. Additionally, the higher Kappa value for FAST + MRI indicates stronger diagnostic consistency with the surgical/pathologic gold standard.

In clinical practice, FAST serves as a practical first-line assessment tool that complements cross-sectional imaging. Its bedside use facilitates rapid triage and early decision-making, particularly before higher-resolution imaging such as MRI is performed. When combined with MRI, FAST serves as an initial triage and screening tool, whereas MRI provides high-resolution anatomical detail and superior soft-tissue contrast for characterizing solid-organ and hollow-viscus injuries. Although our study did not directly evaluate management changes, this sequential FAST + MRI strategy may influence clinical decision-making by helping to select operative versus non-operative treatment, tailor the intensity of monitoring, and reduce repeat CT use in hemodynamically stable patients, particularly when CT is contraindicated, unavailable, or inconclusive.

Previous study has confirmed that early and accurate diagnosis is very important for abdominal cavity organ injury (such as gastrointestinal tract and bladder) caused by closed abdominal trauma, which is directly related to the treatment strategy and survival prognosis of patients (15). In this study, the accuracy of FAST combined with MRI in locating and diagnosing abdominal cavity organ injuries was found to be higher than that of CT. This improved accuracy may be attributed to the fact that injuries to hollow organs, such as the gastrointestinal tract, jejunum, colon, and bladder, can present as subtle tears or perforations. CT images are frequently compromised by bone artifacts and gas, which can obscure these small injuries. In contrast, MRI provides superior soft tissue visualization and is not affected by artifacts commonly seen in CT imaging, thereby allowing for more accurate detection of subtle injuries. This study demonstrated that the diagnosis time using FAST combined with MRI was comparable to that of CT, indicating that this combined approach maintains an efficient diagnostic timeframe for evaluating closed abdominal trauma. The reported FAST + MRI diagnostic times reflect optimized workflows under controlled study conditions, and may not be achievable in typical emergency settings where MRI access is limited and clinical prioritization differs. Consistent with findings reported by Mao et al. (16), who noted improved detection rates for prostate cancer using a combination of ultrasound and MRI, this study similarly highlights the diagnostic synergy achieved by pairing FAST’s rapid preliminary assessment with MRI’s detailed anatomical evaluation. This sequential approach optimizes the overall diagnostic workflow and enhances the accuracy of injury detection in blunt abdominal trauma.

This study demonstrated that FAST + MRI achieved higher diagnostic accuracy than CT, although it did not assess whether this diagnostic improvement affected patient outcomes. The absence of outcome data such as treatment response, complication rate, or hospital stay duration should be considered when interpreting the findings. The analysis was limited to diagnostic accuracy, and therefore, the conclusions apply strictly to imaging performance.

These findings should be interpreted as hypothesis-generating and applicable only to a narrow clinical context. Specifically, our study cohort was limited to hemodynamically stable patients without contraindications to MRI, which restricts the generalizability of the FAST + MRI approach to broader trauma populations. We do not propose replacing CT in standard trauma workflows; rather, our results support the potential value of FAST + MRI as a complementary strategy in select cases where CT is unavailable, inconclusive, or contraindicated. Additionally, the study did not include injury grading, vascular injury assessment, or correlation with treatment interventions such as surgery or interventional radiology, which further limits the comprehensiveness of our findings. Future prospective studies with broader inclusion criteria are needed to determine whether these results can be extended to routine clinical practice.

While our results suggest that FAST combined with MRI improves diagnostic accuracy compared to CT, the cost-effectiveness of this approach requires further evaluation. MRI is generally more expensive and less accessible than CT, especially in emergency settings. Although MRI provides superior soft-tissue evaluation, its longer scan time, higher cost, and requirement for patient cooperation limit its use in emergency settings. It is therefore not universally applicable to critically ill or unstable trauma patients, and FAST + MRI should be considered only in selected hemodynamically stable individuals or when CT is contraindicated or inconclusive. Therefore, the additional diagnostic value of MRI must be weighed against the higher costs and associated logistical challenges. Comprehensive cost-effectiveness analyses and prospective, outcome-driven clinical trials will be essential to inform evidence-based adoption of this imaging strategy in routine trauma care.

Our findings should also be interpreted within their methodological constraints. The study cohort included only hemodynamically stable patients without contraindications to MRI, limiting generalizability to broader trauma populations. Additionally, some CT examinations were non-contrast, which may have influenced the comparative performance results. The study did not include injury grading, vascular injury assessment, or treatment correlations such as surgery or interventional radiology outcomes.

To improve clarity and avoid redundancy, we have now condensed all study limitations into a single, concise paragraph near the end of the Discussion.

These findings should be interpreted within the limitations of this study. The research was conducted at a single tertiary care center with a relatively small and selective sample, limited to hemodynamically stable patients without contraindications to MRI. Approximately one-fifth of CT examinations were non-contrast, which may have reduced CT’s diagnostic performance and influenced the comparative results. Additionally, the study did not include injury grading, vascular injury assessment, or correlation with treatment outcomes such as surgery or interventional radiology. The reliance on follow-up rather than surgical confirmation in some cases may also introduce verification bias. Finally, the retrospective design may have introduced selection bias and limits causal inference.

Despite these constraints, the study demonstrates that in hemodynamically stable patients, the combination of FAST and MRI provides superior diagnostic accuracy and localization compared with CT. FAST + MRI can therefore be considered a complementary imaging strategy when CT is inconclusive or contraindicated, although further validation in larger and more diverse trauma populations is warranted.

In summary, in hemodynamically stable patients, FAST combined with MRI demonstrated higher diagnostic accuracy in our selected patient group, but further validation is needed before its clinical integration, particularly in comparison to contrast-enhanced CT. This combined approach effectively diagnoses both abdominal parenchymal and abdominal cavity organ injuries while maintaining an efficient diagnostic timeframe. Nevertheless, the findings are hypothesis-generating and should be interpreted with caution due to methodological limitations. Our findings indicate that in hemodynamically stable patients, FAST + MRI may serve as a useful complementary imaging strategy to CT; further prospective studies comparing FAST + MRI with contrast-enhanced CT and incorporating clinical outcomes are essential to validate the utility of this imaging strategy.

## Data Availability

The raw data supporting the conclusions of this article will be made available by the authors, without undue reservation.

## References

[1] Abraha D Gebreyes E Wolka E Dender G Sorsa A Muhumuza J. Determinants of adverse management outcomes of blunt abdominal trauma patients operated at a referral hospital in southern Ethiopia: a retrospective record review. BMC Surg. (2023) 23:357. doi: 10.1186/s12893-023-02261-7, 37990208 PMC10664474

[2] Cioffi SP Cimbanassi S Chiara O. Blunt abdominal trauma: watch and wait. Curr Opin Crit Care. (2023) 29:674–81. doi: 10.1097/MCC.0000000000001095, 37861213

[3] Mukharjee S Dinesh BV Bharath SV. Evaluation of management of CT scan proved solid organ injury in blunt injury abdomen—a prospective study. Eur J Trauma Emerg Surg. (2024) 50:2753–63. doi: 10.1007/s00068-024-02501-2 38512418

[4] Chughtai T Parchani A Strandvik G Verma V Arumugam S El-Menyar A . Trauma intensive care unit (TICU) at Hamad General Hospital. Qatar Med J. (2019) 2019:5. doi: 10.5339/qmj.2019.qccc.5 PMC7003060 32076594

[5] Sliwinski S Bechstein WO Schnitzbauer AA Malkomes PTZ. Penetrating abdominal trauma. Chirurg. (2020) 91:979–88. doi: 10.1007/s00104-020-01272-x, 32945917

[6] Gaasch SS Kolokythas CL. Management of intra-abdominal traumatic injury. Crit Care Nurs Clin North Am. (2023) 35:191–211. doi: 10.1016/j.cnc.2023.02.011, 37127376

[7] Patel DD Shih-Della Penna DC Terry SM. Splenic trauma from colonoscopy: a case series. Int J Surg Case Rep. (2020) 71:30–3. doi: 10.1016/j.ijscr.2020.04.057, 32428829 PMC7235943

[8] Yates JG Baylous D. Air medical ultrasound: looking back to see what we have learned for the future. Air Med J. (2022) 41:536–41. doi: 10.1016/j.amj.2022.08.006, 36494169

[9] Sharobaro VI Ivanov YV Sharobaro VI Smirnov AV. Abdominal pseudohernia: diagnosis and treatment. Khirurgiia. (2021):72–80. doi: 10.17116/hirurgia202112172 34941212

[10] Wang Y Li H. Application value of CT in qualitative diagnosis and localization diagnosis of closed abdominal trauma. Chin J CT MRI. (2019) 17:87–9.

[11] Sargent W Bull AMJ Gibb I. Focused assessment with sonography in trauma (FAST) performance in paediatric conflict injury. Clin Radiol. (2022) 77:529–34. doi: 10.1016/j.crad.2022.04.001, 35469663

[12] Rajput MZ Mellnick VM. The role of magnetic resonance in evaluating abdominopelvic trauma—part 2: trauma in pregnancy, vascular, and genitourinary injuries. Can Assoc Radiol J. (2022) 73:689–96. doi: 10.1177/08465371221077654, 35282712

[13] Ibrahim A Wales PW Aquino MR Chavhan GB. CT and MRI findings in pancreatic trauma in children and correlation with outcome. Pediatr Radiol. (2020) 50:943–52. doi: 10.1007/s00247-020-04642-z, 32172401

[14] Keymeulen A De Leenheer E Casaer A Cossey V Herregods N Laroche S . Cranial ultrasound and MRI: complementary or not in the diagnostic assessment of children with congenital CMV infection? Eur J Pediatr. (2022) 181:911–20. doi: 10.1007/s00431-021-04273-y, 34636957

[15] Wackerly R Thomas K Loomis T Moeller D Loomis M. Anatomical correlation for focused assessment with sonography in trauma. Cureus. (2023) 15:e37714. doi: 10.7759/cureus.37714, 37206498 PMC10191455

[16] Mao RD Williams TP Shah NR Snyder C Person J Klimberg VS . Remote instruction in focused assessment with sonography in trauma (FAST) exams for surgery residents: a pilot study. Am Surg. (2023) 89:5407–13. doi: 10.1177/00031348231157821, 36789639

